# Efficacy and safety of the Latarjet procedure for the treatment of athletes with glenoid bone defects ≥ 20%: a single-arm meta-analysis

**DOI:** 10.1186/s13018-024-04641-y

**Published:** 2024-03-01

**Authors:** Ling Wang, ShengRong He, Xia Wu, XiaoYu Lv, Tao Wang, HongBo Tan

**Affiliations:** 1 People’s Liberation Army Joint Logistic Support Force 920th Hospital, Kunming, 650100 China; 2 The Third Affiliated Hospital of Kunming Medical University (Yunnan Cancer Hospital/Yunnan Cancer Center), Kunming, 650100 China; 3 The Third People’s Hospital of Kunming, Kunming, 650041 China

**Keywords:** Latarjet, Athletes, Glenoid bone defect, meta-analysis

## Abstract

**Background:**

The shoulder joint is the most commonly dislocated joint in the human body, and the recurrence rate exceeds 50% after nonsurgical treatment. Although surgical treatment reduces the recurrence rate, there is controversy regarding the optimal surgical approach. Previous studies suggest that the Latarjet procedure yields favourable outcomes for specific populations at risk of recurrence, such as competitive athletes with significant glenoid defects. However, most of the existing related research consists of nonrandomized controlled trials with small sample sizes, and there is a lack of strong evidence regarding the efficacy and safety of the Latarjet procedure.

**Methods:**

The PubMed, Embase, Cochrane Library, and Web of Science databases were systematically searched. Athletes with ≥ 20% glenoid defects were selected for inclusion. The following data were extracted: general patient information, instability rates, return to sports (RTS) rates, imaging features (graft positioning rate and graft healing rate), functional assessments [Rowe score, Athletic Shoulder Outcome Scoring System(ASOSS), visual analogue scale (VAS), forward flexion function, and external rotation function], and complications.

**Results:**

After excluding suspected duplicate cases, a total of 5 studies were included in this meta-analysis. The studies involved a total of 255 patients, including 237 males (93%) and 18 females (7%). The average age at the time of surgery was 25.4 ± 8.5 years. All the studies had a minimum follow-up period of 2 years, with an average follow-up time of 48.7 ± 18.9 months. The pooled rate of return to sport (RTS) was 94.3% (95% CI: 87.3%, 98.8%), and 86.1% (95% CI: 78.2%, 92.5%) of patients returned to their preoperative level of activity. The pooled redislocation rate was 1.1% (95% CI: 0%, 3.8%). Regarding the imaging results, the combined graft retention rate was 92.1% (95% CI: 88.1%, 95.5%), and the graft healing rate was 92.1% (95% CI: 88%, 95.4%). Postoperative functional evaluation revealed that the combined Rowe score, ASOSS score, and VAS score were 93.7 ± 6.5 points, 88.5 ± 4.4 points, and 1.1 ± 10 points, respectively. The forward flexion and external rotation angles were 170.9 ± 6.9 degrees and 65.6 ± 4.5 degrees, respectively. After excluding one study with unclear complications, the combined complication rate was 9.4% (95% CI: 1.0%, 23.6%).

**Conclusion:**

For athletes with shoulder instability and a total of ≥ 20% glenoid bone defects, the Latarjet procedure can achieve excellent functional outcomes, with the majority of patients returning to preoperative levels of sports activity. This procedure also leads to a low recurrence rate. Therefore, the Latarjet procedure has been proven to be a safe and effective treatment.

## Introduction

The shoulder joint, which has unique anatomical and biomechanical characteristics, is highly susceptible to instability and is the most commonly dislocated joint in the human body, with a dislocation rate exceeding 50% [1]. More than half of patients with shoulder instability experience recurrence after nonsurgical treatment [2]. This recurrent dislocation not only imposes limitations on daily activities but also significantly correlates with the progression of osteoarthritis [3]. Common surgical procedures include the Bankart and Latarjet procedures. Of these, the Bankart repair is the procedure of choice for most anterior shoulder instabilities, and it has yielded favourable outcomes in numerous studies [4–6]. However, for athletes at high risk of recurrent shoulder instability, such as those with ≥ 20% glenoid bone defects, the Bankart repair is still associated with a notable risk of recurrence [5, 7–9]. Therefore, the selection of surgical procedures necessitates careful consideration of relevant risk factors [10].

In 1954, Michel Latarjet introduced an open surgical technique involving the transfer of the coracoid to the anterior edge of the glenoid known as the Latarjet procedure [11]. This bone-blocking procedure boasts clear advantages in patients with severe glenoid bone defects [12]. In addition to restoring shoulder stability, early return to sports (RTS) is pivotal, especially for athletes. Nevertheless, the Latarjet procedure is not without complications, with reported complication rates ranging from 7–30% [13–15]. Furthermore, a considerable number of patients face challenges in RTS postoperatively [16]. While several clinical trials suggest that the Latarjet procedure results in lower recurrence rates and excellent clinical outcomes for athletes with glenoid defects greater than 20%, almost all similar studies are nonrandomized controlled trials with small sample sizes; therefore, there is a lack of strong evidence regarding the efficacy and safety of the Latarjet procedure for specific populations [17–21]. Thus, this meta-analysis aimed to assess the effectiveness and safety of the Latarjet procedure for treating athletes with glenoid bone defects ≥ 20% and anterior shoulder instability. These findings will provide robust statistical evidence for clinical application.

## Method

This meta-analysis was reported in accordance with the Preferred Reporting Items for Systematic Reviews and Meta-Analyses (PRISMA) guidelines [21].

### Search strategy

On November 6, 2023, we systematically searched the PubMed, Embase, Cochrane Library, and Web of Science databases. Our search utilized MeSH terms and free-text keywords, specifically “Athlete” and “latarjet”. The language was restricted to English, with no limitations regarding publication date. Additionally, we scrutinized the reference lists of the included literature and early reviews to ensure that any studies overlooked during the electronic database searches were included.

### Inclusion and exclusion criteria

The inclusion criteria for inclusion in this meta-analysis were as follows: (1) had a glenoid bone defect ≥ 20%, (2) had undergone any Latarjet surgical intervention (including classical the Latarjet procedure and other modified procedures), and (3) had an average follow-up time of 2 years or more. The exclusion criteria were as follows: (1) involved other conditions, such as fractures requiring intervention; (2) the sample size was fewer than 10 participants; and (3) included animal experiments, cell studies, reviews, meta-analyses, duplicates, case reports, or letters.

### Literature selection

Two authors independently screened the titles and abstracts of all identified studies from the initial search, and they excluded irrelevant literature. Subsequently, both authors meticulously reviewed the full texts of the remaining studies to determine their eligibility. Any discrepancies were resolved through open discussion with other authors.

### Data extraction and quality assessment

The following data were extracted: the study design, publication year, patient count, basic demographic information, follow-up time, surgical technique, measurement method for glenoid bone defects, and comprehensive details on functional outcomes and complications. The major outcomes extracted included instability and recurrence rates, with secondary outcomes encompassing the radiologically confirmed graft integration rate, graft healing rate, Rowe score, Athletic Shoulder Outcome Scoring System(ASOSS), visual analogue scale (VAS), forward flexion, external rotation angle measurements, and occurrence of complicationss. The collected data were duplicated and meticulously organized in a Microsoft Excel spreadsheet. The risk of bias assessment was performed using the Joanna Briggs Institute (JBI) evaluator manual, consistent with orthopaedic standards.

### Statistical analysis

All variable data in this meta-analysis were analysed using Stata 18 (StataCorp). Chi-square tests and I² tests were employed for heterogeneity assessment, where *p* < 0.1 indicated statistical significance. In the presence of significant heterogeneity (*p* < 0.1 and I²> 50%), random effects models were used for analysis; otherwise, fixed effects models were applied [22].

## Results

### Literature search and study characteristics

**Fig. 1:**
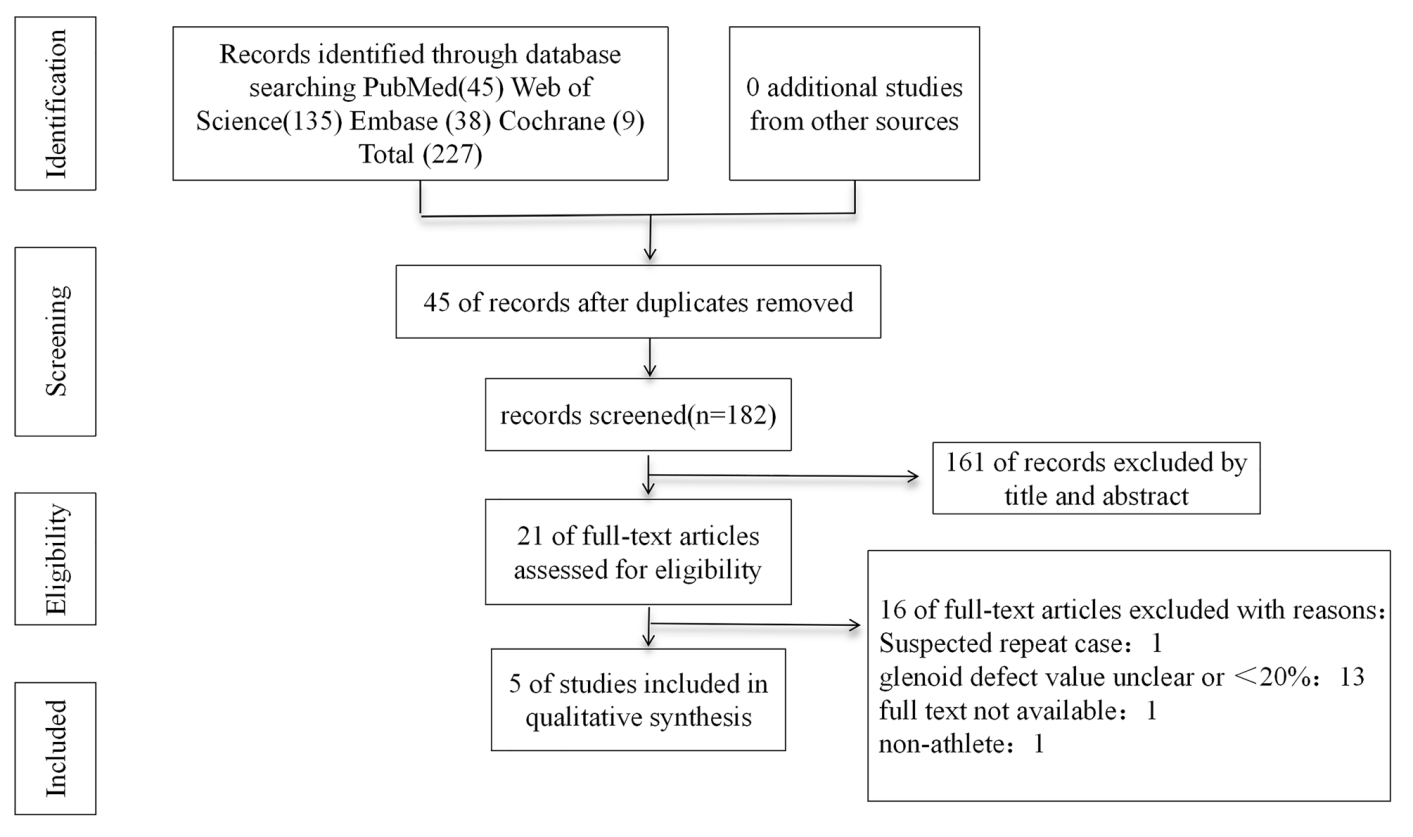
Flow diagram of the meta-analysis for the inclusion/exclusion of studies

An initial search across four databases (PubMed = 45, Embase = 38, Web of Science = 135, and Cochrane Library = 9) yielded 227 relevant published studies. After excluding 45 duplicate studies using Endnote20, two independent researchers screened the titles and abstracts of the remaining 182 articles, excluding 151 irrelevant studies. Subsequently, a thorough assessment of the remaining 21 articles led to the exclusion of 16 publications due to the unavailability of full texts, noncompliance with the inclusion criteria, or suspected duplicate cases. Finally, five articles [17–21] were included in the meta-analysis, and the PRISMA selection flowchart is shown in Fig. 1.

A total of 5 studies were included in this meta-analysis; these included 3 retrospective case series and 2 retrospective comparative studies. The combined sample size of the 5 studies was 392 patients. Since all the studies were conducted at the same medical institution in Argentina, we carefully examined the characteristics of each case and identified 137 suspected duplicate cases. Despite our efforts to contact the authors for additional data, we were unable to obtain the necessary information. To ensure the accuracy of our analysis, we excluded the suspected duplicate cases and ultimately included 255 patients in this meta-analysis. Among these patients, 237 (93%) were males and 18 (7%) were females. The average age at the time of surgery was 25.4 ± 8.5 years. All the studies had a minimum follow-up period of 2 years, with an average follow-up time of 48.7 ± 18.9 months. Table 1 provides an overview of the key characteristics of the included studies.

**Table 1:** Basic characteristics of the included studies

Study name	Local	Sample size, n	Follow-up, mouth	Age, year	Dominant, n	Sex(male/female, n)	Revision procedures, n	Glenoid bone loss, %
Brandariz 2021	Argentina	29	35 ± 6	32.3 ± 10.1	20	27/2	12	/
Ranalletta 2018	Argentina	65	47.2 ± 17.9	28.3 ± 8.7	36	63/2	65	28.2 ± 4.2
Rossi 2018	Argentina	46	59.4 ± 18.9	26.7 ± 6.5	26	40/6	0	25.5 ± 3.6
Rossi 2020	Argentina	55	36.4 ± 5.0	27.2 ± 5.7	35	50/5	55	26.4 ± 3.2
Rossi 2022	Argentina	60	60.1 ± 20.7	16.4 ± 1.0	27	57/3	28	25 ± 2.1

For clinical outcome assessment, all five studies reported rates of redislocation, RTS occurrence, radiologically confirmed graft healing, Rowe score, ASOSS score, forward flexion, and external rotation function scores [17–21]. Four studies reported graft integration rates [17, 19–21], four reported VAS scores [18–21], and four provided information on complications [17–19, 21], with three studies noting complications [17, 19, 21]. One study lacked detailed complication descriptions for the analysed subgroups and was thus excluded from the complication analysis [20].

### Quality assessment results

The risk of bias assessment was performed using the Joanna Briggs Institute (JBI) evaluator manual, which comprises ten items evaluating the quality of case reporting, including case selection, disease or health problem assessment, and case data presentation. The quality assessment details are available in Table 2.

**Table 2:** Quality Assessment of the Studies Included in the Meta-Analysis

Study	Q1	Q2	Q3	Q4	Q5	Q6	Q7	Q8	Q9	Q10
Rossi 2022	Y	Y	Y	Y	Y	Y	Y	Y	Y	Y
Brandariz 2021	Y	Y	Y	Y	Y	Y	Y	Y	Y	Y
Ranalletta 2018	Y	Y	Y	Y	Y	Y	Y	Y	Y	Y
Rossi 2018	Y	Y	Y	Y	Y	Y	Y	N	Y	Y
Rossi 2020	Y	Y	Y	Y	Y	Y	Y	Y	Y	Y

Numbers Q1-Q10 in heading signified: Q1, were there clear criteria for inclusion in the case series? Q2, was the condition measured in a standard, reliable way for all participants included in the case series? Q3, were valid methods used for identification of the condition for all participants included in the case series? Q4, did the case series have consecutive inclusion of participants? Q5, did the case series have complete inclusion of participants? Q6, was there clear reporting of the demographics of the participants in the study? Q7, was there clear reporting of clinical information of the participants? Q8, were the outcomes or follow-up results of cases clearly reported? Q9, was there clear reporting of the presenting site(s)/clinic(s) demographic information? Q10, was statistical analysis appropriate?Y: Yes, N: No

### Analysis results

#### Rate of instability

**Fig. 2:**
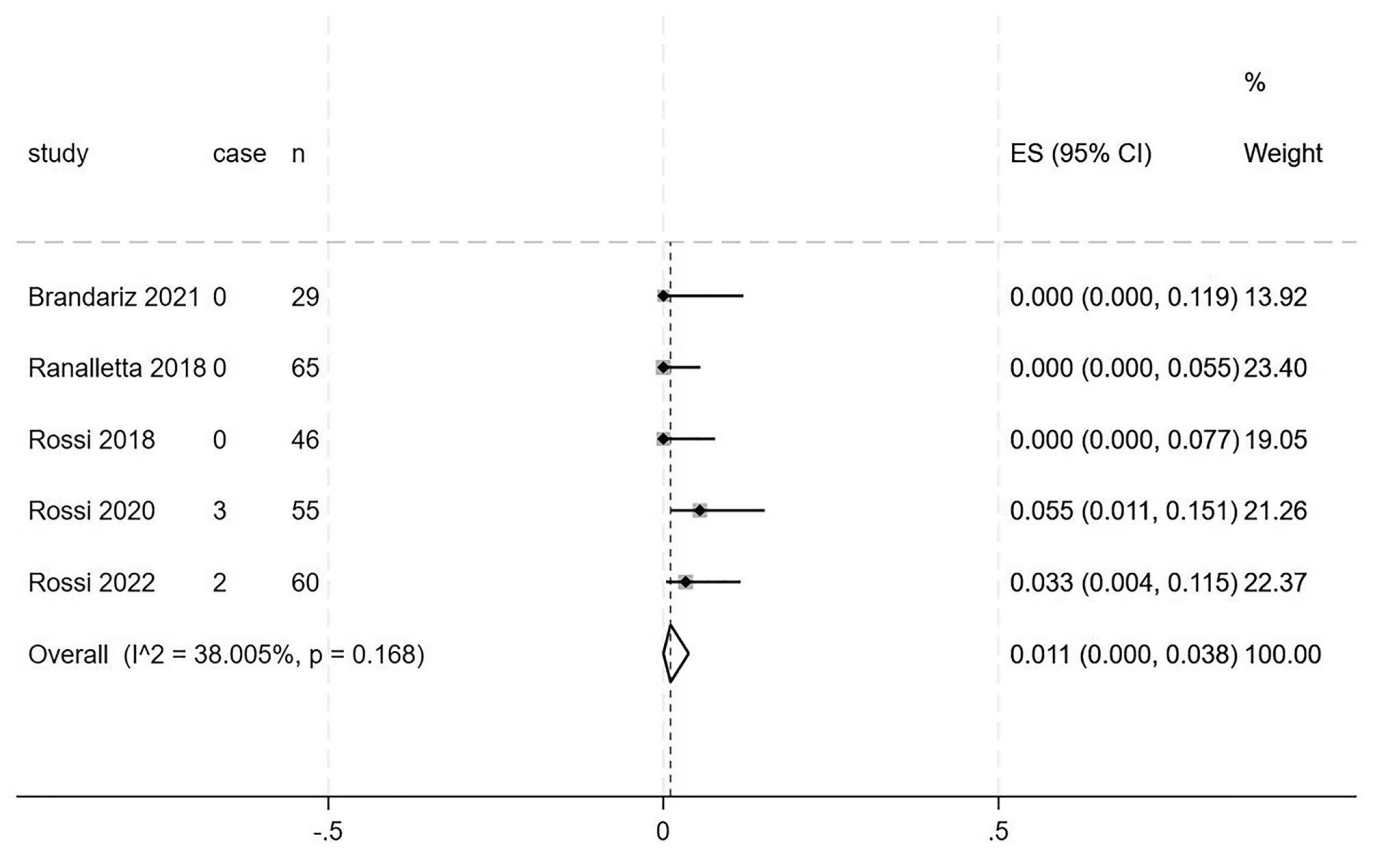
Forest plot illustrating the rate of postoperative redislocation in patients following the Latarjet procedure

All five studies included in this meta-analysis, encompassing a total of 255 patients, reported postoperative redislocation information [17–21]. There was a low level of heterogeneity between the studies (I^2^ = 38.00%, *p* = 0.168); thus, a fixed effects model was used. The redislocation rate was 1.1% (95% CI = 0%, 3.8%), as shown in Fig. 2.

### Return to sports

**Fig. 3:**
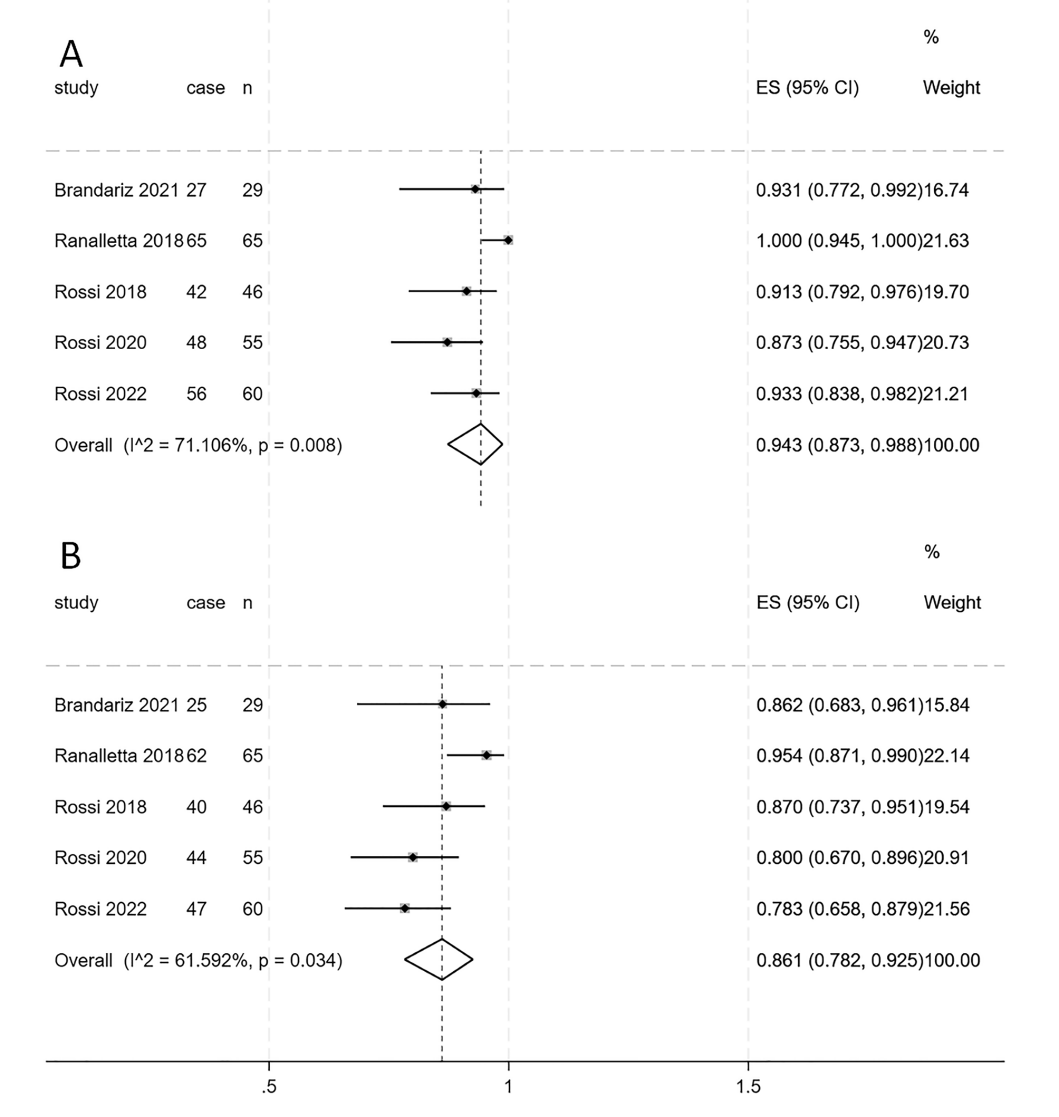
**A**, Forest plot depicting the rate of return to sports in patients following the Latarjet procedure; **B**, Forest plot illustrating the proportion of patients who returned to preoperative levels of physical activity after the Latarjet procedure

All five studies included in this meta-analysis, comprising 255 patients, reported information related to RTS after the Latarjet procedure [17–21]. A random effects model was used (I²=71.1%, *p* = 0.008), and the combined RTS rate was 94.3% (95% CI 87.3%, 98.8%), as shown in Fig. 3A. A random effects model was applied to the proportion of patients who recovered preoperatively (I²=61.5%, *p* = 0.034), revealing a combined proportion of 86.1% (95% CI = 78.2%, 92.5%), as shown in Fig. 3B.

### Radiological outcomes

**Fig. 4:**
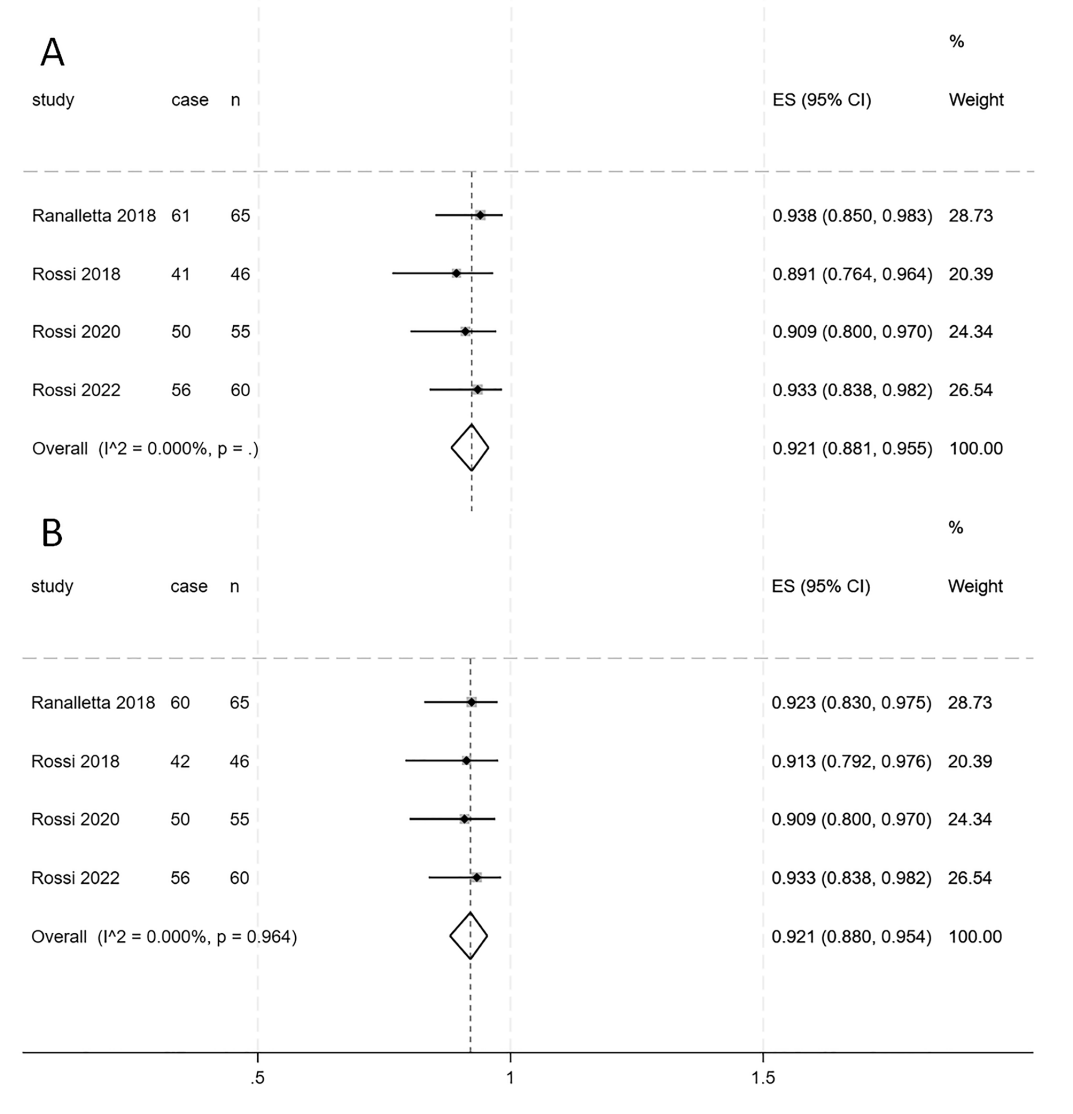
**A**, Forest plot depicting the graft position on CT scans at a follow-up of at least 3 months post surgery; **B**, Forest plot illustrating the graft healing rate on CT scans at a follow-up of at least 3 months post surgery

The radiological outcomes primarily included graft integration rates and graft healing rates, as shown by CT scans at least three months after surgery. Four studies involving 226 patients reported graft integration rates after the Latarjet procedure [17, 19–21], with no heterogeneity observed between studies (I²=0%, *p* = 0.000). The combined postoperative graft integration rate was 92.1% (95% CI 88.1%, 95.5%), as illustrated in Fig. 4A. All four studies, encompassing 226 patients, reported graft healing rates [17–21], with no heterogeneity observed between studies (I²= 0%, *p* = 0.000). The combined graft healing rate for 255 patients postoperatively was 92.1% (95% CI 88%, 95.4%), as displayed in Fig. 4B.

### Functional outcomes

**Fig. 5:**
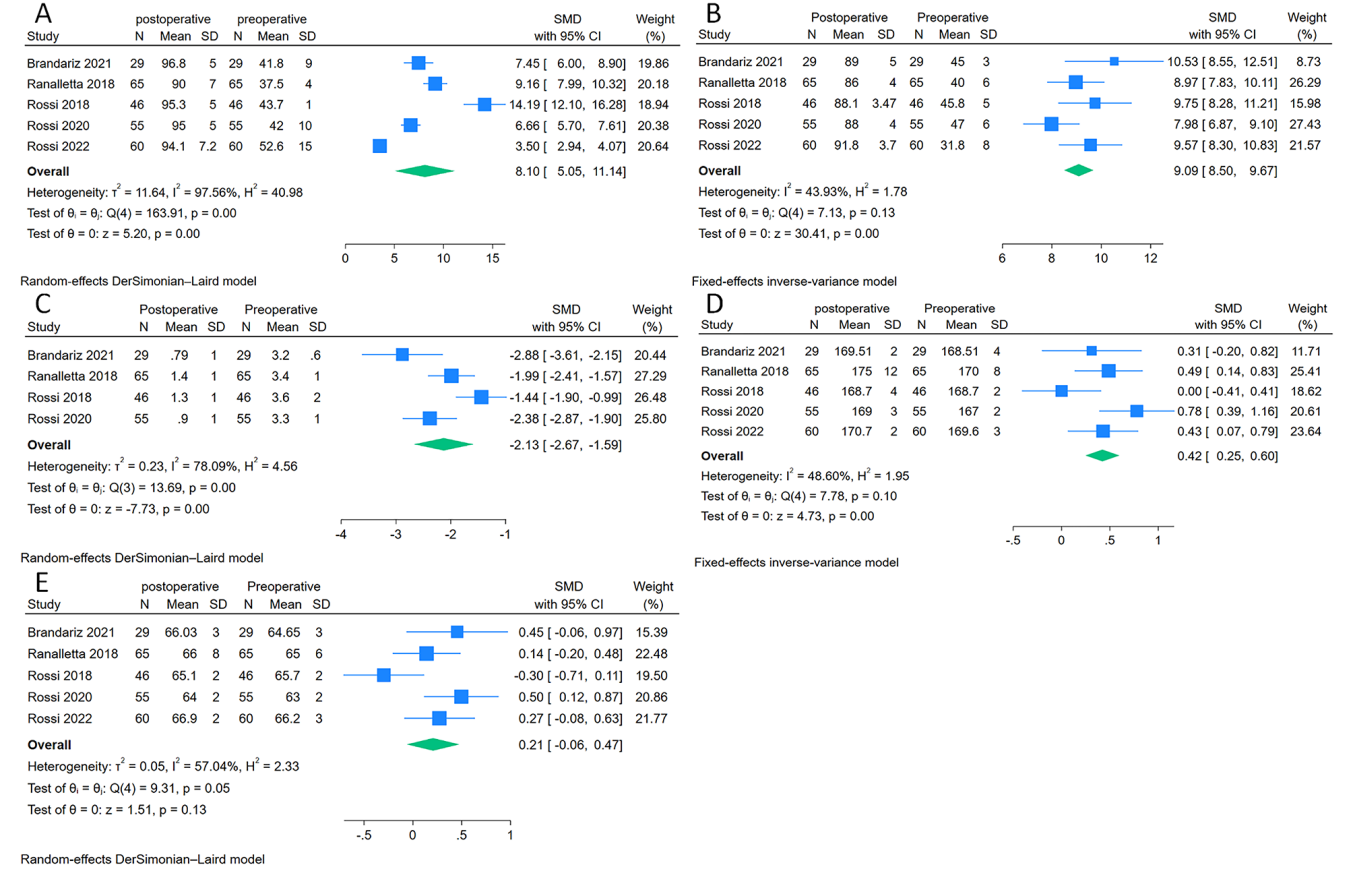
**A**, Forest plot of Rowe scores before and after surgery; **B**, Forest plot of ASOSS scores before and after surgery; **C**, Forest plot of VAS scores before and after surgery; **D**, Forest plot of preoperative and postoperative forward flexion function scores; **E**, Forest plot of preoperative and postoperative external rotation function scores

All five studies included in this meta-analysis involving 255 patients reported Rowe scores, ASOSS scores, forward flexion function, and external rotation function scores [17–21]. Four studies involving 195 patients reported VAS scores [18–21]. The data were extracted at two time points: before surgery and at the latest follow-up. During the meta-analysis, patients were divided into two groups according to postoperative functional outcome scores: the experimental group and the control group. The results showed significant improvements in postoperative Rowe scores, ASOSS scores, VAS scores, and forward flexion function scores compared to preoperative scores. Rowe scores were influenced by heterogeneity (I²= 97.56%, *P* = 0.00) according to the random effects model (SMD = 8.10;95% CI 5.05, 11.14; *P* = 0.00); ASOSS scores showed low heterogeneity between studies (I²= 43.93%, *P* = 0.13) according to the fixed effects model(SMD = 9.09; 95% CI(8.50, 9.67; *P* = 0.00); VAS scores were influenced by heterogeneity (I²= 78.09%, *P* = 0.00) according to the random effects model (SMD=-2.13; 95% CI -2.67, -1.59, *P* = 0.00); forward flexion function scores exhibited low heterogeneity between studies (I²= 48.60%, *P* = 0.10) according to the fixed effects model (SMD = 0.42; 95% CI 0.25, 0.60; *P* = 0.00). The external rotation function score was influenced by heterogeneity (I²=57.04%, *P* = 0.05) according to the random effects model, with no significant difference between the two groups (SMD = 0.21; 95% CI -0.06, 0.47; *P* = 0.13), as shown in Fig. 5. The average Rowe score, ASOSS score, and VAS score at the latest follow-up were 93.7 ± 6.5, 88.5 ± 4.4, and 1.1 ± 10, respectively. The forward flexion and external rotation angles were 170.9°±6.9° and 65.6°±4.5°, respectively.

### Complications

This study examined the postoperative complications of the Latarjet procedure in athletes with unstable shoulder joints and glenoid bone defects ≥ 20%. One study with unclear complications was excluded from the analysis. The overall incidence of complications was 9.4% (95% CI: 1.0%, 23.6%) based on a total of 27 reported cases from 4 studies. Among the 5 patients who experienced recurrence of instability, 2 underwent revision surgery with autologous iliac crest grafts, while 3 received physical therapy; all of these patients were able to return to their presurgical exercise levels. Additionally, two patients reported postoperative shoulder pain, which resolved after arthroscopic screw removal. Four patients developed postoperative infections, all of whom responded well to treatment. Specifically, one patient with intra-articular infection was treated with open lavage, while the other three patients with superficial infection were treated with oral antibiotics. Furthermore, 13 new cases of arthritis were observed after surgery, including 10 cases of mild arthritis and 3 cases of moderate arthritis. No instances of severe arthritis were documented across any of the studies. Graft fragmentation, haematoma, and musculocutaneous neuropraxia each occurred in one patient, and all patients showed improvement following conservative treatment. Detailed information about the complications can be found in Table 3.

**Table 3:** Complications of the studies included in the meta-analysis

Complications	ES, %(95 CI)	I^2^
instability	1.5%(0–5.1%)	44.39%
Pain	0.6%(0%,2.6%)	0
Infection	1.7%(0.1%,4.2%)	0
hematoma	0.2%(0%,1.7%)	0
Myocutaneous nerve disorders	0.1%(0%,1.7%)	0
Arthritis	3.9%(0%,13.6%)	83.44%
Graft fragmentation	0.2%(0%,1.7%)	0
Total	9.4%(1.0%, 23.6%)	86.57%

## Discussion

The most significant finding of this study is that the Latarjet procedure results in excellent functional outcomes and a low recurrence rate for athletes with ≥ 20% glenoid bone defects and anterior shoulder instability. This finding was substantiated by statistical evidence.

The primary objective of restoring patient stability is to prevent the recurrence of instability and facilitate patient return to normal life and work. Athletes, in particular, face heightened demands on shoulder stability and function due to increased physical and competitive stress. Previous research has demonstrated that soft tissue repair surgeries in high-risk athletes are associated with increased recurrence risk [5, 8, 23–25]. However, the postoperative instability rate after the Latarjet procedure varies, ranging from 1–16% [26–29], with a 1.1% recurrence rate in this study. The heterogeneity among studies may be attributed to significant differences in baseline characteristics between studies; for example, Lima et al. [29] focused on females with relatively low shoulder instability rates, while females composed only 7% of the participants in this study. In contrast, clinical study results on RTS rates generally agree. A systematic review revealed that 83% of athletes who underwent bone reconstruction surgery were able to undergo RTS [30]. Hurley et al. [31] evaluated the efficacy of the Latarjet procedure for shoulder instability over a 10-year follow-up and reported an RTS rate of 84.9%, with 76.3% of patients returning to their preoperative activity level. Rassoul et al.‘s [5] systematic review comparing arthroscopic the Bankart repair, arthroscopic Bankart repair with Remplissage (REMP), arthroscopic Latarjet procedure, and open Latarjet procedure showed that open the Latarjet procedure had a similar RTS rate (83.6%), but this rate was significantly lower than that of the arthroscopic Bankart repair (97.5%), REMP (95.5%), and arthroscopic Latarjet procedure (94.0%). Davis et al.‘s [32] study suggested that REMP is superior to simple Bankart repair or the Latarjet procedure, with an RTS rate of 86%. In our study, the postoperative RTS rate was 94.3%, with an average of 86.1% of patients returning to their preoperative activity level. This result is slightly greater than that of previous studies and is similar to the findings of Horinek et al. [33], who considered REMP and the Latarjet procedure to have similar outcomes in patients with shoulder instability and > 15 glenoid defects.

Hurley et al. [31] evaluated the clinical efficacy of the Latarjet procedure for shoulder instability over a 10-year follow-up and reported an average Rowe score of 88.5. Horinek et al. [34] assessed VAS scores and forward flexion and external rotation angles and reported average VAS scores of 2.2 preoperatively and 1.3 at the last follow-up; forward flexion angles of 171° preoperatively and 178° at the last follow-up; and external rotation angles of 64° preoperatively and 82° at the last follow-up. Rossi et al. [35] evaluated ASOSS scores in a cohort study of athletes and reported an average preoperative ASOSS score of 53.1 and an average score of 93.7 at the last follow-up. The specific reasons for the difference in external rotation function are unknown, but there is a noticeable difference in measuring 0° abduction passive external rotation and 90° abduction passive external rotation. However, existing studies do not provide detailed descriptions. Current research suggests that the Latarjet procedure may result in a decrease of approximately 5° in the external rotation angle, necessitating proactive rehabilitation exercises postoperatively [28].

The other results in our study align closely with previous ones. The majority of patients achieved good to excellent scores, indicating satisfactory functional outcomes for this patient group. Additionally, biomechanical studies suggest a negative correlation between the incidence of glenoid defects and shoulder joint stability, with a glenoid defect of 20% considered a critical threshold for recurrence in Bankart repair treatment for shoulder instability [36, 37]. Like glenoid defects, shoulder instability in young athletes is a risk factor for postoperative recurrence after surgery [23]. Therefore, the population included in this meta-analysis had relatively greater risk factors for recurrence than did the population included in the aforementioned studies.

The occurrence rate of adverse events after the Latarjet procedure is significant, with Mizuno et al. [38] reporting a 20% incidence of bony arthritis changes in an average 20-year follow-up after the Latarjet procedure. Recently, Hurley et al. [31] conducted a systematic review evaluating the long-term risk of bony arthritis after the Latarjet procedure and reported a 38.2% incidence of arthritis changes in patients followed for at least 10 years. In this study, with a minimum follow-up time of 2 years, the combined incidence of new arthritis was 3.9%,with no evidence of severe bony arthritis changes. Other adverse events included graft nonhealing, haematoma, and infection, consistent with previous research [28, 39–41]. While earlier studies provided high-quality research evidence through long-term follow-up, a series of changes, including surgical instruments and continued education stemming from long-term follow-up, also impact postoperative functional outcomes. In this study, the Latarjet procedure for athletes with glenoid bone defects ≥ 20% and anterior shoulder instability demonstrated favourable functional outcomes.

Despite these notable findings, this study has certain limitations. First, there was considerable heterogeneity between the included studies, particularly in terms of baseline patient characteristics. Second, the studies included in this analysis had small sample sizes and lacked control groups. As a result, our assessment focused primarily on efficacy and risk, with no definitive evidence establishing the superiority of the Latarjet procedure over alternative interventions. Additionally, despite the consistent use of assessment methods in the included literature to mitigate bias, the homogeneity in the geographic origin and medical institution of all studies, coupled with a relatively limited sample size and predominantly male patients, necessitates caution in extrapolating these results to broader populations. Consequently, larger-scale randomized controlled trials should be meticulously designed to confirm the clinical efficacy of the Latarjet procedure compared with other interventions across diverse patient cohorts.

## Conclusion

In summary, this meta-analysis provides robust evidence supporting the Latarjet procedure’s exceptional functional outcomes for athletes with glenoid bone defects ≥ 20%. The majority of patients exhibited a successful return to sports preoperatively, and there was a low recurrence rate, suggesting that the Latarjet procedure is a safe and effective therapeutic option in clinical practice. However, due to the inherent limitations of limited clinical data, future research should include multicentre randomized controlled trials featuring extended follow-up periods to substantiate and refine these findings.

## Data Availability

No datasets were generated or analysed during the current study.
